# Supplementary material for: How do researchers approach societal impact?
Source: PLoS One. 2021 Jul 9;16(7):e0254006. doi: 10.1371/journal.pone.0254006 (PMC8270479; doi:10.1371/journal.pone.0254006)
Supplement: Attachment — Submitted filename: response_PONE-D-21-03474.docx [file pone.0254006.s001.docx]

Berlin, May 6 2021

Dear colleagues,

We would like to thank the reviewers very much for this most helpful and competent review. The comments were consistently intelligible to us and formulated in a way that we were able to implement them.

In particular, we have (1) added substantially to the literature on societal impact and its evaluation, (2) added an analysis section and elaborated on the conceptual framework, (3) rewrote the introduction and discussion sections. We furthermore adapted the tables and figures and had the entire manuscript professionally proofread. Please find below a table, in which we address each of the points you mentioned in the review.

We hope that we have been able to address your feedback to your satisfaction and would also be pleased if we could remain in collegial exchange beyond this publication.

Yours sincerely,

Benedikt Fecher & Marcel Hebing

| PONE | |
| --- | --- |
| (A) Please ensure that your manuscript meets PLOS ONE's style requirements, including those for file naming. | We did revise the manuscript accordingly. |
| (B) Please improve statistical reporting and refer to p-values as "p<.001" instead of "p=.000". | Done. |
| (C) Please include a copy of Table 3 which you refer to in your text on page 19. | Table 3 is no longer required and we removed the reference from the text. |
| (D) Please ensure that you refer to Figure xxxxx in your text as, if accepted, production will need this reference to link the reader to the figure. | We did revise the manuscript accordingly. |
| Reviewer 1 | |
| (1) The whole analysis section is missing regarding the statistical analysis. The reader cannot see how the authors conducted the study. There is only one section where the authors uses a regression analysis, while the whole analysis is descriptive. It needs a good reason to ground such an analytical choice. The statistical description requires re-phrasing and the tables need reformulations. It is rather difficult to understand the authors’ writing through these expressions and the table. | Agreed. We added an analysis section, describing the statistical approach (p. 15f). We furthermore added a conceptual framework, explaining our operationalization of societal impact as well as the explanatory dimensions (p. 7f). Please note that the reason why we conducted a primarily descriptive analysis is that the topic is hardly empirically examined from the researchers’ perspective and we aimed to explore the topic, giving an overview of the status quo. This procedure is also quite common, also in other PLOS publications (see for example Tenopir (2011): Data Sharing by Scientists: Practices and Perceptions). We made this point clearer in several parts of the manuscript. We furthermore updated all the tables and figures (p. 14, p. 17, p. 18f, p. 20, p. 23f). |
| (2) Because the questionnaire of this research is self-designed, there should be a description of its validity and reliability, which are now missing. | We conducted two rounds of pretests to evaluate the validity and reliability of the instrument. We added a section on the pretests (p. 12). We furthermore extended the part on the qualitative pre-study in the method section, which is the basis for the questions parts on RQ2 and RQ3 (p. 11). |
| (3) Because the method, analysis and results are insufficiently presented, I strongly recommend asking an expert in statistics to re-check and revise these sections. I believe such expertise would help to improve the readability, validity and reliability of this article. | We completely reworked the method section (p. 10), added an analysis section (p. 15f). Furthermore, we condensed the results section (p. 16f.). We had statistic experts in the first pretest round and had a statistics expert reading the revised manuscript. We hope that with these changes, we were able to significantly enhance the readability and overall comprehensibility of the manuscript. |
| (4) The theoretical framework relating to science communication in the manuscript does not connect to the results of the study. The interpretation of the results lacks a meaningful theoretical framework, although the study presents a framework for science communication. I recommend to structure a hypothesis vis-à-vis science communication and interpret the results by this framework for the study. | We added a conceptual framework that explains the operationalization of societal impact (p. 7f) and the explanatory dimensions (p.8). We relate to Bornmann (2013) when it comes to the operationalization of societal impact as well as De Jong (2014) to explain how societal impact is linked to (science) communication. We believe that the connection of the science communication discourse (in communication science) and the societal impact discourse (in evaluation research) is a worthwhile approach to understand societal impact. We agree, however, that this was not necessarily evident in the previous version of the article. Please note: We present the results (p. 16f) vis-à-vis the research questions and in the individual results sections, we use our explanatory dimensions to structure the report. We hope that this makes the presentation and interpretation of the results much clearer. |
| (5) There are several theoretical approaches to social impact of research or science communication and the authors should point out what point of view their hypothesis and results are supporting. The discussion section lacks correspondence to academic discussion about research impact. | We added substantially to the section on the scholarly discourse on societal impact (p. 6f) and furthermore added an operationalization for our approach (p. 7f) in the paper. We furthermore added substantial sources in the introduction section (p. 2f), the literature review (p. 4f) and in the discussion (p. 25f). Overall, we think that due to these amendments and additions, the manuscript is much more coherent. Thank you! |
| (6) I recommend that a professional language service would proofread this article. | The last version of the manuscript was reviewed by a professional proofreading service. The language should be clean. |
| (7) The title of the paper corresponds weakly to the study, as the paper studies people’s opinions and understanding of their engagement, not how they actually achieve impact. | Agreed. We modified the title, which is now: “How do researchers approach societal impact?” |
| (8) The study utilizes the categories of the Research Excellence Framework (REF) from the United Kingdom. However, the REF cannot be argued to be without normative assumptions when it is a result of policies. The utilization in analysis needs some theoretical justification for using such criteria for impact. | Please note, that we did not use exactly the categories from the REF but generated the categories through a qualitative coding of the REF impact case studies. We furthermore added a few sentences on the impact case studies and explain that these are of course not without normative assumptions (p. 11f.). |
| (9) Basic and applied distinction is not always clear and divergent disciplines may have differing opinions about this. Use of such distinctions needs theoretical support. | The distinction between applied and basic research is based on the subjective self-assessment of the respondents. While it is correct that opinions differ, the same holds true for other subjective self-assessments in surveys of this type. One of the reasons for the regression analysis was that it enables us to control for disciplines while analysing this distinction. |
| (10) The questionnaire has a section considering respondent’s personality. I cannot see this data in the paper and the meaning of such data for the paper. | In this article we report on the major part of our survey, but there are still parts in the questionnaire which we don't report on here because they are intended for other projects. We added more details in the instrument section to clarify this approach (p. 11f). Fyi: The part of the survey on personality and data sharing is to revise a previous study we published at PLOS: https://journals.plos.org/plosone/article?id=10.1371/journal.pone.0183216 |
| (11) What do the authors mean by: "...by the socio-demographic factors: gender, status, and age."? In addition what “status”, do you mean employment status? Please, be more specific of these terms. | The term "status" refers to the academic status of the participants. We reviewed the entire article to be specific and consistent in the use of this term. |
| (12) Expression “In relation to the engagement with society” is unclear. It can be read that research is not part of society. | We rewrote the parts about engagement. Specifically on p. 7 we make clear that science is part of society. |
| (13) Did you select the universities according to your criteria for the selection among all universities in Germany? | We selected the universities and applied universities based on the number of students and chose the 20 largest and the 20 smallest as well as 20 medium-sized ones in Germany. In each case, we contacted every faculty head with the request to distribute the survey. Additionally, we contacted the directors of each institute within the biggest German nonuniversity research organizations, i.e., the Max Planck Society, the Leibniz Association, the Helmholtz Association, and the Fraunhofer Gesellschaft. We also contacted the German Research Association’s (DFG) graduate schools. We made the sample design and distribution clearer in the manuscript (p. 13). |
| (14) Your description of a semi-convenient/convenient sample is not consistent in the paper. | We revised the use of the term and checked that it is used consistently. |
| (15) The result section is rather long, and some parts are quite repetitive. Shortening it could make it more reader-friendly. | We have tried to make the wording of the results more coherent and thus shorten the section. Nevertheless, the results presented seem consistently relevant to us, which is why we have not removed actual results. |
| (16) Description of the REF coding needs to be placed in the method section. | The description of the REF coding is now part of the method section (p. 11f). |
| (17) Table 2 title needs to be more specific. | We reviewed all captions of figures and tables and made them more informative, including table 2. |
| (18) Table 3 is missing completely. | We removed table 3 to not overload the paper with tables. Unfortunately, we forgot to remove the reference in the text (which is now deleted). |
| (19) What does “sustainable strategy for impact” mean in this context? | We rephrased this. What we meant is that, in line with the meta-scientific discourse on evaluation, impact agendas need to take epistemic conditions into account. |
| (20) What is specific impact in relation to disciplines? I don't think reference to a policy paper is relevant in discussion part. It should have discussion with the science communication literature or impact literature. | We rewrote the whole discussion section (p. 25f). It is now more closely aligned with the research questions and makes reference to current research, including your paper in Science and Public Policy (which we read with great interest). |
| Reviewer 2 | |
| (21) In the section SOCIETAL IMPACT IN RESEARCH p 10 ff you may problematize and position your study more by (i) a deeper description existing research of the different concepts of academic knowledge creation see e.g., Olsson et al 2020, (2020). A conceptual model for university-society research collaboration facilitating societal impact for local innovation. European Journal of Innovation Management.<https://doi-org.ezproxy.server.hv.se/10.1108/EJIM-04-2020-0159> and (ii) earlier research on measuring and defining research impact e.g., De Jong, et al (2014) “Understanding societal impact through productive interactions: CT research as a case”, Research Evaluation, Vol. 23 No. 2, pp. 89-102; Greenhalgh, T., Jackson, C. et al (2016), “Achieving research impact through co-creationin community-based health services: literature review and case study”, The Milbank Quarterly, Vol. 94 No. 2, pp. 392-429; Matsumoto, M., et al (2010), “Development of a model to estimate the economic impacts of R&D output of public research institutes'', R & D Management, Vol. 40 No. 1, pp. 91-100. Issues of co-creation in university-society collaboration is also something you may find interesting see e.g., Larsson, J. and Holmberg, J. (2018), “Learning while creating value for sustainability transitions: the case of challenge lab at Chalmers university of technology”, Journal of Cleaner Production, Vol. 172, pp. 4411-4420, and Olsson et al 2020 as above. | Thank you very much for the valuable literature references! We elaborated the part on societal impact in research, relating to the sources you mentioned and others as well (p. 6f). We furthermore added a section on our operationalization of societal impact (p. 7f). Here, we use De Jong et al.’s notion of “productive interactions” for justifying our explanatory variables. |
| (22) AIM – there is a slight difference between the aim on page 5 and page 19. RQ1 includes the words engagement, evaluation and press departments that need further explanation and linked to earlier research in the introduction section. This is also reoccurring in the Method section p. 7 as the importance of communication and performance of communication department- is there any earlier research on these aspects then it should be mentioned in the Introduction. | We rewrote the respective sections. We furthermore added sources to the literature review, that explain why we included items regarding evaluation and communication to the opinion items. Specifically, regarding the research on communication departments, we added research (p. 5f). |
| (23) p. 6 define status in the individual dimension. | The term "status" refers to the academic status of the participants. We reviewed the entire article to be specific and consistent in the use of this term.  See (11). |
| (24) P. 12 The status groups -presented differently throughout the paper. A logical presentation of the groups is recommended along with an early definition in the paper of how you define a ‘researcher’. | The survey addressed anyone who considered him- or herself an academic researcher. By asking the participants about their academic status, discipline, institutional type, etc. we hope that we were able to give a more detailed overview of who our participants (=researchers) were. See in particular p. 14f (sample description). |
| (25) The age of 18 seems to be very young to be included in the group of researchers? | For legal reasons, we did not want to address minors. So instead of defining the smallest age group as something like "< 29 years" we made it more explicit by writing "18-29 years", also we didn't expect respondents of age 18 as well. |
| (26) Methodology and results are presented and illustrated in a proper way. | Thank you. :) |
| (27) Fig 1 Institutional types- may the categories be merged into three categories instead? | We are not sure if we understand this feedback correctly. Could you rephrase that, please? |
| (28) The manuscript ends with a Discussion – add Conclusion in the heading or add another subsection entitled Conclusion. There are very few references in your Discussion. Return to your earlier refences in the Introductions section and compare your findings to earlier research. | We rewrote the entire discussion and conclusion. We renamed this section to “Discussion and Conclusion”. We added sources and returned to our earlier references. Thank you for this hint! It makes the article more coherent. |
