# Supplementary material for: How do researchers approach societal impact?
Source: PLoS One. 2021 Jul 9;16(7):e0254006. doi: 10.1371/journal.pone.0254006 (PMC8270479; doi:10.1371/journal.pone.0254006)
Supplement: Attachment — Submitted filename: Response_to_reviewers_PONE-D-21-03474.docx [file pone.0254006.s002.docx]

| RESPONSE TO REVIEWER | |
| --- | --- |
| Reviewer 2 | |
| Pretests: The authors explain that they have done pretests for reliability, but they do not elaborate what result the test provided for reliability, though they explain what they did based on those tests. | (1) The pretests revealed issues regarding the wording of those questions that we designed ourselves. In most cases, we had used words that were either specific to particular disciplinary fields / institutions or simply ambiguous. Here are some examples:   - “Press department” → “communication department” (Question C1) - “Health of population” → “wellbeing of the population” (C3) - “Civil society” → “organized civil society” (C4)   (2) Furthermore, some questions (in particular C1) were almost twice as long (twice the number of items to answer) in the beginning. The pretests helped us to identify less promising items to shorten survey. The expert feedback was that the shorter survey was significantly easier to answer, resulting in a higher quality of responses.  → We included a short description of both kinds of updates in the article (p.12f). We hope this solves the issue. |
| In the sample design, the authors describe that they selected the universities based on number of students. Why do they not rely it on researchers, teachers and other staff of scholars when the study is about researchers’ attitudes? | We agree that the number of researchers would have been a better basis for choosing universities. However, there is only robust data for “personnel at universities” in destatis (i.e. administrative staff included). We decided that the number of students is a better proxy for choosing universities to contact.  We added a short explanation in the text (p.13):  “The reason for selecting universities and universities of applied sciences by number of students (rather than number of researchers) is explained by the availability of better statistical data.” |
| On page 19, the expression sounds ambiguous: “Fewer researchers, but still many,…” | This refers to the section summary of the results. The numbers are reported above. However, we agree that the statement sounds ‘fuzzy’. Therefore we added the numbers for agreement. Thanks for pointing that out! |
| In the results section (RQ 1) the authors conclude that: “It is noteworthy that researchers (especially at universities) do not think that knowledge transfer plays an important role at their institution and that institutional communication departments are reaching relevant societal stakeholders. From this, we infer that societal impact is understood as an individual task for which there is no institutional mandate.  It is a bit unclear in this argument whether it means understanding of these institutions or the understanding of the researchers in the survey. If this means only the research staff’s attitudes, I suggest to formulate the conclusion differently, so that it is clear that it describes only the staff’s believes. Of course these two dimensions could be compared preferably.  This conclusion of neglected organizational aspects of impact requires information about the actual knowledge transfer activities of the survey institutions, and how much emphasis they have on impact evaluation and practices. Now the authors provide information about general policy recommendations and considerations, but this does not describe what kind of practices the universities have regarding impact requirements, their organization and evaluation, and their relationship with the survey attitudes.  Perhaps, the authors could provide some basic information of how impact is considered in the university and research work in Germany. | Agreed. We added “from the perspective of the researchers in our survey” to that statement to emphasize that this mirrors the researchers’ perceptions (p. 20).  In the discussion of the results we further differentiate this finding (p. 28f).  Thanks for the pointer. This differentiation is important! |
| The claim “but these were chosen by less than 10% of the respondents and are therefore excluded” is mentioned twice in the same context. | We deleted this note in the figure description and left it in the text. Thanks! |
| In the results (RQ2), the authors claim that “It is striking that the disciplinary groups have quite different impact profiles: Scholars from the humanities tend to have culture- and discourse-oriented goals, social scientists have discourse-, social-justice-, and policy-oriented goals, and natural scientists have technology-, health-, and environment-oriented goals.  This claim is one of the main results in the paper. However, there is nothing striking in this argument according to common knowledge of disciplinary practices and recent studies on research impact and public engagement. Perhaps, the authors could emphasize how these findings support previous understanding of the characteristics of these disciplines and their public engagement. | Well, it is “striking” in the very neutral meaning of the term. However, you are right that this is common knowledge and has been addressed before, although – to our knowledge – not in a differentiated and empirically-based manner. We agree that these findings might therefore add to the ongoing discourse.  We decided to leave the wording in the respective part of the text and to expand on the contextualization of the finding in the discussion, including relevant sources (p.27 f). |
| Then the authors conclude that: “From this, we conclude that societal goals are best explained by the disciplinary backgrounds of researchers.”  Because several authors have already pointed out this argument in recent literature (e.g. Benneworth) and in older classics (e.g. Weiss), the authors could elaborate this statement by linking it to recent literature, which is worried about impact policies neglecting such disciplinary goals and their importance. | See above. Agreed. |
| The results (RQ3) bring out education offerings as an important perception of impact. I was wondering why this does not seem to consider the education of the institutions themselves, as they provide a great number of new graduates for employment each year. | In the survey we particularly asked for “education offerings, e.g. in schools” (as we write in the results). However, in the question on societal goals (RQ2), we asked for education in general, on the assumption that contributions to education can be a societal goal. For transparency reasons, we added a sentence there (p. 21):  “In this regard, it should be noted that we did not explicitly ask about non-university education on the assumption that university teaching (as well as contributions to non-academic education) can be understood by the respondents as a society-oriented goal.” |
| Usually some sort of limits of the study should be brought out in the conclusions. |  |
| The conclusions seem a bit unfinished. Maybe the main conclusion is that disciplines have had their own impact goals in relation to contemporary society all along, and now all the responsibilities are suddenly pressured against researchers without proper institutional structures. On the other hand, what are the impact goals of the organizations and do they correspond to disciplines’ goals? As I pointed out in the sixth comment, linking the findings to recent studies on the disputes about impact goals and neglection of disciplinary goals, to older discussion about the difference between research utilization of various academic fields and to the structural/organizational levels of research impact could benefit and increase the value of the paper. | We added substantially to the discussion and conclusion. In particular, we   1) better relate our findings to the sources in the background (e.g., when it comes to disciplinary differences, the findings reg. the humanities, the notion of dialogue as a prerequisite for impact) 2) reflect the findings with the policy parts in the introduction (e.g., the BMBF’s call for broad impact vs. the alliance’s call for differentiated impact) 3) contextualize the organizational findings for the German context (e.g., reg. the third mission strategies of German universities)   Thank you for this comment; we hope that our changes add to the quality of the conclusion. |
| The authors point out: certain mismatch between individual and institutional commitment. However, what are the actual institutional demands from individuals? | Good point! We made this clearer throughout the text, a) by adding literature on impact and third-mission strategies in Germany and b) by softening this statement (e.g., p.20: “From this, we infer that—from the perspective of the researchers in our survey—societal impact is understood as an individual task for which there is no institutional mandate.”). |
| I find this interesting: "Humanities scholars especially use social media to communicate results."  Perhaps, the authors could speculate a bit why the survey has such a surprising result. | We added an interpretation and relevant sources (p.28): “Humanities scholars especially use social media to communicate results (55% compared to 33% of natural scientists and 38% of social scientists). One explanation could be that, as shown above, humanities scholars tend to be discourse-oriented and social media are a suitable tool to achieve this goal [61,90].”  [61. Benneworth P. Tracing how arts and humanities research translates, circulates and consolidates in society.. How have scholars been reacting to diverse impact and public value agendas? Arts Humanit High Educ. 2015;14: 45–60. doi:10.1177/1474022214533888](https://www.zotero.org/google-docs/?PdqbwW)  [90. Holmberg K, Thelwall M. Disciplinary differences in Twitter scholarly communication. Scientometrics. 2014;101: 1027–1042. doi:10.1007/s11192-014-1229-3](https://www.zotero.org/google-docs/?PdqbwW) |
| This is unclear: researchers have teaching obligations not faced by those at independent institutes and thus less time for engagement activities. However, researchers from applied universities typically have a higher teaching workload.  What does this mean regarding engagement activities? | Researchers at independent institutes do not have to do any teaching which gives them arguably more time for engagement. For some independent instituts, transfer is the main mission (in particular Fraunhofer institutes).  We made the particular sentence clearer and also want to point out that we explained the differences between the institutional types in more depth in the method section. We hope this suffices. |
| I find this statement unnecessary, as the paper clearly has a normative tone: While our results are mainly of a descriptive nature and we do not make normative assumptions about the subject of our research (i.e. “it is good to have more impact… | You are right. We deleted that part of the sentence. |
| “The findings further suggest that, where applicable, organizational factors (e.g., institutional investments in transfer, training offerings, support infrastructures) should be more strongly incorporated into assessments of societal impact”  I don’t understand how the authors came to this conclusions. Why should they be part of assessments of impact? Why is impact assessment the perfect way to promote public engagement? There are also other ways to organize and promote knowledge in society. | There must be a misunderstanding: Of course, there are other ways to promote impact, some of which we delineate (e.g., support structures, disciplinary discussions, etc.). We do also not argue for evaluation in general, but rather that when evaluations are carried out, they should not only look at the effects of single formats, but also at the conditions for impact. We have tried to make this point clearer by briefly describing formative methods and by referring to the BMBF's policy paper (which suggests summative procedures) in the introduction to the discussion. In other parts of the paper, we furthermore made clear that the results are based on researchers’ perceptions. We hope, this solves this. |
| This gives a misleading impression that innovation is same than impact: “In addition, we suggest that national innovation systems should be studied comparatively in order to understand the impact of policies and to study more closely the relationship between transfer practices and societal impact in specific disciplines.” | We adapted that sentence. It is now “research systems”. |
| Some minor notions:  - “An example of this is Jong et al.'s concept of productive interactions…”  Productive interactions is a concept of Spaapen and van Drooge (2011).  - “According to Bornmann, three main strands of societal impact can be distinguished:”.  This is probably de Jong et al. (2014) interpretation based on Bornmann’s conceptualization.  - The instrument: “Nevertheless, as researchers’ expressions of impact, they provide a suitable textual data basis for developing goal and format categories”  Some studies (Watermeyer et al.) have shown that the REF case studies construct unsophisticated imaginaries of impact. This problematics could be mentioned regarding the evaluation competition. | - We adapted the literature. Thanks for spotting this! - We added a limitation paragraph, mentioning Watermeyer |
